# An explicit Wiener–Hopf factorization algorithm for matrix polynomials and its exact realizations within ExactMPF package

**DOI:** 10.1098/rspa.2021.0941

**Published:** 2022-07

**Authors:** V. M. Adukov, N. V. Adukova, G. Mishuris

**Affiliations:** ^1^ Institute of Natural Sciences and Mathematics, South Ural State University, Chelyabinsk 454080, Russia; ^2^ Institute of Mathematics, Physics and Computer Science, Aberystwyth University, Aberystwyth SY23 3BZ, UK

**Keywords:** matrix polynomials, Wiener–Hopf factorization, Toeplitz matrices, essential polynomials, exact computation, implementation

## Abstract

We discuss an explicit algorithm for solving the Wiener–Hopf factorization problem for matrix polynomials. By *an exact solution* of the problem, we understand the one constructed by a symbolic computation. Since the problem is, generally speaking, unstable, this requirement is crucial to guarantee that the result following from the explicit algorithm is indeed a solution of the original factorization problem. We prove that a matrix polynomial over the field of Gaussian rational numbers admits the exact Wiener–Hopf factorization if and only if its determinant is exactly factorable. Under such a condition, we adapt the explicit algorithm to the exact calculations and develop the ExactMPF package realized within the Maple Software. The package has been extensively tested. Some examples are presented in the paper, while the listing is provided in the electronic supplementary material. If, however, a matrix polynomial does not admit the exact factorization, we clarify a notion of the numerical (or approximate) factorization that can be constructed by following the explicit factorization algorithm. We highlight possible obstacles on the way and discuss a level of confidence in the final result in the case of an unstable set of partial indices. The full listing of the package ExactMPF is given in the electronic supplementary material.

## 1. Introduction and motivation

The factorization problem has proven to be an extremely efficient tool in mathematics (for example, in the theory of linear operators [1,–3]) and instrumental in various branches of physics, mechanics and engineering (see, for example, [4,–8]).

Unfortunately, in contrast with the scalar problems, factorization of arbitrary matrix functions remains an unresolved problem [9,10] (especially, when one speaks of its effective applications). We start with definitions, preliminary results and than formulate the problem considered in this paper.

Let Γ be a simple piecewise smooth closed contour in the complex plane C bounding the domain $D_{+}$. We can assume that $0\in D_{+}$. The complement of $D_{+}\cup \Gamma$ in the closed complex plane, $\bar{C}=C\cup \{\infty \}$, is denoted by $D_{-}$. Let a(t) be p×p matrix function on Γ. In what follows we assume that a(t) is continuous and invertible on Γ.

The representation of a matrix function a(t)

1.1 $$a(t)=r_{-}(t)d_{R}(t)r_{+}(t),\;t\in \Gamma ,$$

is called *the right Wiener–Hopf factorization* of the matrix function [1]. Here, $r_{\pm }(t)$ are continuous and invertible matrix functions on Γ that admit analytic continuation into $D_{\pm }$ and their continuations $r_{\pm }(z)$ are invertible into the respective domains; matrix function $d_{R}(t)=\text{diag}\;[t^{\rho_{1}},\ldots ,t^{\rho_{p}}]$, where integers $\rho_{1},\ldots ,\rho_{p}$ are called *the right partial indices* of a(t). It is always possible to assume that $\rho_{1}\leq \ldots \leq \rho_{p}$. Moreover, $\rho_{1}+\cdots +\rho_{p}=\varkappa$, where ϰ=ind deta(t) is the winding number (or the Cauchy index) of the determinant of the matrix a(t).

Similarly, *the left Wiener–Hopf factorization* of a(t) is the representation [1]

$$a(t)=l_{+}(t)d_{L}(t)l_{-}(t),\;t\in \Gamma ,$$

where $l_{\pm }(t)$ have the same properties as $r_{\pm }(t)$, $d_{L}(t)=\text{diag}[t^{\lambda_{1}},\ldots ,t^{\lambda_{p}}]$, and $\lambda_{1}\geq \cdots \geq \lambda_{p}$ are called *the left partial indices* of the matrix function a(t) ($\lambda_{1}+\cdots +\lambda_{p}=\varkappa$). Note that, if the factorizations exist, their left and right indices are usually different sets of integers and constructions of the right and left factorizations are usually considered as two separate problems. On the contrary, a method proposed in this work requires simultaneous considerations of both factorizations.

In general, a matrix function a(t) with continuous entries does not admit the Wiener–Hopf factorizations with factors in the same class. It is known that if the elements of a(t) satisfy the Hölder condition on Γ or belong to the Wiener algebra W(T), where T is the unit circle in the complex plane, then the Wiener–Hopf factorizations exist (see [1]).

The scalar factorization problem for a non-singular function a(t) can always be solved explicitly, see, for example, [11] and [12]. Unfortunately, explicit formulae for the factors $r_{\pm }(t)$, $l_{\pm }(t)$ and the partial indices of an arbitrary matrix function do not exist at present.

Fortunately, there are classes of matrix functions for which an *explicit (or constructive) solution* to factorize the problem have been found. By this, we understand that there are explicit formulae allowing us to formally construct the partial indices and the factors of the matrix function a(t). For example, the problem for matrix polynomials has been solved explicitly [–1,13,15]. For meromorphic matrix functions, there also exits an explicit solution [13]. A detailed review of constructive methods for the factorization problem is presented in the work [10] (see, also [16]).

Unfortunately, the factorization problem for matrix functions is generally speaking unstable. More precisely, if the difference between the larger and the smaller partial indices is greater than one, then the set of the partial indices can change with an arbitrary small perturbation of the initial matrix [12,17]. Moreover, if left (right) factorization appears to be stable, this does not mean that the right (left) preserves that property [1]. There is also a technical difficulty embedded into the problem as the factors $r_{\pm }$, $l_{\pm }$ are not found uniquely, while how to properly normalize the algorithm to guarantee the uniqueness in the chosen class is still open. On the other hand (see, for example, [18], theorem 6.15), if two matrix functions a(z) and $\tilde{a}(z)$ are close enough, and their partial indices coincide, then it is always possible to find a factorization of $\tilde{a}(z)$ for which factors ${\tilde{a}}_{\pm }(z)$ are close enough to the factors of $a_{\pm }(z)$. Recently, a few constructive approximate techniques for factorization of specific matrix functions have been proposed [19,–22], under the condition that their sets of partial indices are known in advance. Accompanied stability analysis has also been performed in the case of unstable partial indices.

Therefore, *the key step* in construction of any (exact or approximate) factorization of a given matrix function (if the factorization exists) is to provide a procedure/formula allowing for calculation of the partial indices. However, even having an explicit route to achieve the goal, it often requires a numerical realization. And here, one may meet with a hidden unexpected obstacle: the procedure can again be unstable. Below we discuss two simple examples highlighting the issue.

Consider two matrix polynomials (the only elements $p_{11}(z)$ are slightly different there):

1.2 $$\begin{array}{rl} & P_{1}(z)=\left(\begin{array}{cc} 2z^{2}-z+\frac{1}{4} & -4z^{2}+2z-1\\ z & z^{3}+z^{2}+z+4 \end{array}\right)\\ \mathrm{and}\; & P_{2}(z)=\left(\begin{array}{cc} 2z^{2}-z+\frac{1}{2} & -4z^{2}+2z-1\\ z & z^{3}+z^{2}+z+4 \end{array}\right). \end{array}\}$$

To factorize explicitly the polynomials by the method developed in [14], we need first to factorize their determinants. For the first matrix function, we have

1.3 $$\det P_{1}=2z^{5}+z^{4}+\frac{21}{4}z^{3}+\frac{21}{4}z^{2}-\frac{11}{4}z+1$$

and the factorization of this polynomial can only be done approximately. Hence, all subsequent steps for the explicit factorization $P_{1}(z)$ may be unstable. To apply the effective criterion of stability for partial indices [23], we have to verify that a certain matrix with float point entries, built using the matrix $P_{1}(z)$, has the full rank. In turn, this is an ill-condition problem itself if the condition number of the matrix is large enough ([24], ch. 2). Thus, within the framework of this particular and well-developed explicit method, we cannot always even determine for sure whether the partial indices are stable or not. All other methods for matrix polynomials known in the literature [15] have the same obstacle when used to factorize the matrix $P_{1}(z)$.

A completely different situation arises with the polynomial $P_{2}(z)$. Now the determinant

1.4 $$\det P_{2}=2z^{5}+z^{4}+\frac{11}{2}z^{3}+\frac{11}{2}z^{2}-\frac{5}{2}z+2$$

also has its roots that can be found only numerically (and thus, approximately). However, it can be presented as a product of irreducible polynomials $\det P_{2}=(2z^{2}-z+1/2)(z^{3}+z^{2}+3z+4)$ and all roots of $2z^{2}-z+1/2$ lie in an interior of the unit circle, while all roots of the polynomial $z^{3}+z^{2}+3z+4$ lie in the domain exterior. We can verify these statements without calculating the roots themselves by using Schur’s test (see §3). Hence,

1.5 $$\det P_{2}(z)=\left(2-\frac{1}{z}+\frac{1}{2z^{2}}\right)z^{2}(z^{3}+z^{2}+3z+4)$$

is the Wiener–Hopf factorization of the determinant, $\det P_{2}$, over the field of the Gaussian rational numbers Q(i), since the coefficients of the matrix polynomial $P_{2}(z)$ belong to this field. In what follows, we show that all steps of the *explicit* factorization algorithm can be performed in the *exact* arithmetic and, as a result, the instability issue does not arise in this case.

These examples demonstrate that

(i) existence of an *explicit* procedure for a particular matrix factorization does not guarantee that it can be successfully used in practice;
(ii) knowledge of exact values of zeros of the polynomial is not an essential feature (useful though) for execution of the *exact* factorization;
(iii) the notions of *explicit, exact and numerical* factorizations should be clearly distinguished.

By the *explicit* (or *constructive*) solution of the factorization problem, we understand a clearly defined algorithmic procedure that should definitely terminate after a finite number of steps. However, when one implements a specific *explicit* factorization algorithm, each consequent step can be executed *exactly* or *approximately (numerically)*.

To avoid the difficulties associated with possible instability, one can chose the input data belonging to the field Q(i) and perform all steps of the *explicit algorithm* in the *exact arithmetic*. In such a case, we find that there exists *an exact solution* of the factorization problem. Unfortunately, as has been discussed above, this resolution is not always available even when in possession of a few different *explicit* algorithms.

If a factorization problem does not admit *the exact* solution, the only choice that remains is to construct a *numerical (approximate)* factorization. The latter, in view of possible instability, may represent a real challenge for execution and all computations should be done with special care and supplemented by accurate analysis of the partial indices preservation and a numerical stability.

*Thus, the key question arising during the factorization of a matrix function is: how to compute the partial indices with a full confidence?*

There are classes of matrix functions for which the partial indices are known in advance. For example, positive definite matrix functions [12] have all their partial indices equal to zero, while, for matrix functions not containing zero in their numerical ranges [25], they are equal (but not necessarily zeros). Such sets of the partial indices are stable with respect to small perturbation of the matrix functions [12], and, as a result, the remaining task in the factorization process (computation of the factors) is less problematic and any appropriately chosen numerical algorithm can be performed and justified by standard techniques.

In this study, we discuss factorization of matrix polynomials on the unit circle T with particular attention to all these details. The structure of the paper is as follows. In §1, we describe a well-known explicit method for constructing a factorization of a matrix polynomial [14]. We formulated this method algorithmically in order to indicate explicitly all the steps that must be taken to construct the factorization. This will be convenient for further attempts to implement our method numerically. However, in this section, we do not consider computational problems related to our method. Therefore, no analysis of the accuracy (forward/backward error) of the explicit algorithm is carried out in this section. The basic tool for this factorization is a method of essential polynomials [26].

In §3, we show that all steps of the *explicit* algorithm for factorization of a matrix polynomial, a(z), over the Gaussian rational field Q(i) is implemented in the *exact arithmetic* if and only if its determinant, deta(z), allows *exact* factorization in this field. The solution of the latter is presented in theorem 3.1. We used representation a scalar polynomial as the product of its irreducible factors and the Schur algorithm to test whether each factor has its roots siting inside (or outside) the unit circle. Further, all steps of the explicit matrix factorization can be performed in the *exact* arithmetic. The algorithm for *exact* evaluation of the essential polynomials is a foundation stone of theorem 3.2 which is the main result of this section. The formal description of this algorithm is rather complicated and will be presented in the separate work.

In §4, we implemented the exact factorization algorithm as the package ExactMPF in the computer algebra system Maple.^1^ This package consists of the following basic procedures: SchurTest, SolverExactPF, ExactMLC, seqTk, NumRank, ExactFEP. The main procedure is SolverExactMP. The purposes of these procedures are shortly described in the same section, while the full listing of the package ExactMPF is given in the *electronic supplementary material*. Several examples of the *exact* factorizations performed with use of the package ExactMPF are presented in §5 with the aim to demonstrate the package abilities and to study peculiarities of the factorization for special classes of matrix functions. Other tests are used to analyse stability of the computations (see examples 5.1 and 5.2). All those and other examples are included in the electronic supplementary material with more details.

In the general case, the explicit algorithm can only be realized approximately. This poses a real challenge for users due to the instability of the problem. Our numerical experiments with ExactMPF have demonstrated that even in the case of unstable sets of partial indices there is a good chance to obtain an approximate factorization. Namely, to accelerate the work of ExactMPF, we provide an option ‘symnum’ into the SolverExactMP. If this option is turn on, the package calculates the ranks of matrices by the singular value decomposition (SVD) method [24,27]. These numerical ranks are used to find partial indices of the initial matrix polynomial. Further calculations are performed in the exact arithmetic. This allows for a verification of the partial indices finding by SVD versus the exact ones: (are the sets of the partial indices the same or not?). It turned out that for many examples, even with unstable sets of the partial indices, such a numerical approach leads to correct results. The partial indices found with the SVD method are called partial τ-indices of the polynomial. However, the question whether or when they do coincide remains open. The opportunity to use SVD for numerical calculations of partial indices in a more general context is discussed in §6. Finally, in the Conclusion, we underline the advances made in this paper and discuss further possible developments and applications.

## 2. An explicit Wiener–Hopf factorization for matrix polynomials

In this section, following the results of the work [14], we present an *explicit* algorithm for the factorization of matrix polynomials. In accordance with our understanding of the notion of *explicit* solution (see the Introduction), we describe below those finite numbers of steps that, after their execution, allows one to construct a factorization. We focus on the algorithmic structure of *this explicit procedure* that should be implemented within a respective numerical code. We would like to emphasize here that this section is not devoted to any study of possible computational problems that may appear during the algorithm implementation into a specific environment.

We start with **Input data**, that is, a matrix polynomial of a degree n, $a(z)=\sum_{j=0}^{n}a_{j}z^{j}$, represented by the set of its consecutive coefficients, $a_{j}\in C^{p\times p}$, j=0,…,n, and p∈N defines the matrix size. We assume that the matrix polynomial, a(z), is *invertible* on the unit circle T.

Step 1. **Construction of the Wiener–Hopf factorization of the scalar polynomial deta(z)**.
  We denote Δ(z)=deta(z). Since Δ(z)≠0, |z|=1, the determinant admits the Wiener–Hopf factorization
  2.1 $$\Delta (z)=\Delta_{-}(z)z^{\varkappa }\Delta_{+}(z)\;\mathrm{and}\;\Delta_{-}(\infty )=1,$$
  relative to T, that is unique with the additional condition at infinity for the polynomial $\Delta_{-}(z)$. In the sequel, we use, in fact, only one of the factors, namely,
  2.2 $$\Delta_{-}(z)=1+\Delta_{1}^{-}z^{-1}+\cdots +\Delta_{\varkappa }^{-}z^{-\varkappa },\;\varkappa ={\text{ind}}_{T}\det a(z).$$
  It should be noted that any explicit factorization algorithm will use the calculation of the determinant of the factorizable matrix function. The authors realize that the complexity for computing the determinant of a matrix of order n is n!. Fortunately, in applications this happens for matrix function of a small order. Moreover, in §3, we use the matrix determinant in conditions of the exact calculations.
  Here, ϰ is the Cauchy index of deta(z). There is Gakhov’s formula for this, which is convenient for numerical calculations. However, in our case, we can find this index by the exact calculations (see theorem 3.1).
Step 2. **Evaluation of a finite set of Laurent coefficients of the matrix function $\Delta_{-}^{-1}(z)a(z)$.**
  Laurent coefficients of the auxiliary rational matrix function $\Delta_{-}^{-1}(z)a(z)$ are computed in terms of matrix coefficients $a_{j}$ of the original matrix polynomial, a(z), and the coefficients of the scalar polynomial, $\Delta_{-}(z)$, defined on the previous step. The rational function $\Delta_{-}^{-1}(z)$ is analytic in a domain |z|>r for some r<1. Let us expand $\Delta_{-}^{-1}(z)$ and $\Delta_{-}^{-1}(z)a(z)$ in the Laurent series
  2.3 $$\Delta_{-}^{-1}(z)=\sum_{j=0}^{\infty }\frac{\delta_{j}}{z^{j}}\;\mathrm{and}\;\Delta_{-}^{-1}(z)a(z)=\sum_{j=-\infty }^{n}c_{j}z^{j}$$
  in this domain. Coefficients $\delta_{j}\in C$ and $c_{j}\in C^{p\times p}$, in the series above, can be found from the following relations:
  2.4 $$\begin{array}{rl} & \delta_{0}=1,\;\delta_{1}=-\Delta_{1}^{-}\\ & \delta_{j}=\left\{ \begin{array}{ll} -\Delta_{j}^{-}-\sum_{k=1}^{j-1}\delta_{j-k}\Delta_{k}^{-}, & 2\leq j\leq \varkappa ,\\ -\sum_{k=1}^{\varkappa }\delta_{j-k}\Delta_{k}^{-}, & \varkappa +1\leq j\leq \varkappa +n, \end{array}\right. \end{array}\}$$
  and
  2.5 $$c_{j}=\left\{ \begin{array}{ll} \sum_{k=0}^{n}\delta_{k-j}a_{k}, & j\leq 0,\\ \sum_{k=j}^{n}\delta_{k-j}a_{k}, & 0n, \end{array}\right.$$
  where the factor $\Delta_{-}(z)$ has been computed on the previous step in (2.2). In what follows, we need only a finite number of the coefficients, $c_{k}$, for k=−ϰ,…,0,…,ϰ.
  Let $c_{-\varkappa }^{\varkappa }:=\{c_{-\varkappa },\ldots ,c_{0},\ldots ,c_{\varkappa }\}$ be the finite sequence of complex p×p matrices which are the Laurent coefficients of $\Delta_{-}^{-1}(z)a(z)$. We form a finite family of the block Toeplitz matrices of finite sizes
  2.6 $$T_{k}=||c_{i-j}|{|}_{\begin{array}{c} i=k,k+1,\ldots ,\varkappa \\ j=0,1,\ldots ,\varkappa +k \end{array}},\;-\varkappa \leq k\leq \varkappa .$$
  The next step is a corner stone in implementation of the *explicit* algorithm proposed in [14].
Step 3. **Finding the ranks and the null spaces for of block Toeplitz matrices $T_{k}$**.
  Let $\ker_{R}T_{k}$ ($\ker_{L}T_{k}$) be the right (left) kernels of the matrices $T_{k}$. Recall that the right and left kernels of a complex block matrix $A\in C^{(n+1)p\times (m+1)p}$ consisting of p×p blocks are defined by the relations $\ker_{R}A=\{x:Ax=0\},\ker_{L}A=\{y:yA=0\}$.
  Let us represent an element $R\in \ker_{R}A$ in a block form $R=\left(\begin{array}{c} r_{0}\\ r_{1}\\ \vdots \\ r_{m} \end{array}\right),\;r_{j}\in C^{p\times 1}$, and define a column-valued generating polynomial in z related to R as $R(z)=r_{0}+r_{1}z+\cdots +r_{m}z^{m}.$
  By $N_{k}^{R}$, −ϰ≤k≤ϰ, we denote the space of generating vector polynomials for vectors in $\ker_{R}T_{k}$. Put $N_{-\varkappa -1}^{R}:=\{0\}$ and let $N_{\varkappa +1}^{R}$ be (2ϰ+2)p–dimensional space of all column-valued polynomials whose degrees are not greater than 2ϰ+1.
  Repeating the same line of reasoning, we define the space $N_{k}^{L}$, −ϰ≤k≤ϰ of the row-valued generating polynomials in $z^{-1}$ for the rows from $\ker_{L}T_{k}$.
  As a result of this step, the set of ranks of the matrices $T_{k}$ defined in (2.6), and bases of the spaces $N_{k}^{R}$, $N_{k}^{L}$, −ϰ≤k≤ϰ, has been computed. All those elements, values and sets are of finite dimensions.
  In general, computing rank and calculation of basis elements of the null-spaces are unstable processes. In our work, we will find the rank and null-space of a matrix with the help of calculation in the exact arithmetic.
Step 4. **Computation of indices and factorization essential polynomials.**
  The main tools for computations of the partial indices and factors in the factorization of matrix functions are the so-called indices and essential polynomials of the sequence $c_{-\varkappa }^{\varkappa }$ (see [14,26]). Below we give necessary definitions and discuss how to compute them.
  By $d_{k}^{R}$, we denote a dimension of the right kernel $N_{k}^{R}$ and introduce the following integers: $\Delta_{k}^{R}=d_{k}^{R}-d_{k-1}^{R}$ for −ϰ≤k≤ϰ+1. A sequence $c_{-\varkappa }^{\varkappa }$ is called *regular* if $\Delta_{-\varkappa }^{R}=0$ and $\Delta_{\varkappa +1}^{R}=2p$. For a regular sequence, we have (see [14,26])
  2.7 $$0=\Delta_{-\varkappa }^{R}\leq \Delta_{-\varkappa +1}^{R}\leq \cdots \leq \Delta_{\varkappa }^{R}\leq \Delta_{\varkappa +1}^{R}=2p.$$
  Since a monotone integer sequence is piecewise constant, then there are 2p integers $\mu_{1}\leq \cdots \leq \mu_{2p}$ such that
  2.8 $$\begin{array}{c} \Delta_{-\varkappa }^{R}=\cdots =\Delta_{\mu_{1}}^{R}=0,\\ ....................\\ \Delta_{\mu_{i}+1}^{R}=\cdots =\Delta_{\mu_{i+1}}^{R}=i,\\ ....................\\ \Delta_{\mu_{\;2p}+1}^{R}=\cdots =\Delta_{\varkappa +1}^{R}=2p. \end{array}$$
  The absence of the jth row here means that $\mu_{j+1}=\mu_{j}$.
  **Definition 2.1.**
  The integers $\mu_{1},\ldots ,\mu_{2p}$ defined by the relations (2.8) are the indices of the sequence $c_{-\varkappa }^{\varkappa }$.
  It turns out that (see [14,26])
  2.9 $$\sum_{j=1}^{2p}\mu_{j}=0.$$
  Furthermore, we define the right essential polynomials of the sequence $c_{-\varkappa }^{\varkappa }$. Note that $N_{k}^{R}$ and $zN_{k}^{R}$ are subspaces of $N_{k+1}^{R}$ as it follows from the definition of the spaces $N_{k}^{R}$. The dimension, $h_{k+1}^{R}$, of the complement $H_{k+1}^{R}$ of $N_{k}^{R}+zN_{k}^{R}$ in $N_{k+1}^{R}$ is equal to $\Delta_{k+1}^{R}-\Delta_{k}^{R}$.
  Then, equations (2.8) imply that $h_{k+1}^{R}\neq 0$ if and only if $k=\mu_{j}$, j=1,…,2p. Moreover, in this case, $h_{k+1}^{R}$ is equal to the multiplicity, $\varkappa_{j}$, of the index $\mu_{j}$. Therefore, for $k\neq \mu_{j}$, we have
  $$N_{k+1}^{R}=N_{k}^{R}+zN_{k}^{R},$$
  and for $k=\mu_{j}$
  2.10 $$N_{k+1}^{R}=(N_{k}^{R}+zN_{k}^{R})⊕H_{k+1}^{R}.$$
  **Definition 2.2.**
  Any polynomials $R_{j}(z),\ldots ,R_{j+\varkappa_{j}-1}(z)$ forming a basis for a complement $H_{\mu_{j}+1}^{R}$ are called right essential polynomials of the sequence $c_{-\varkappa }^{\varkappa }$ corresponding to the index $\mu_{j}$.
  As a result, we have defined 2p indices $\mu_{1},\ldots ,\mu_{2p}$ and 2p right essential polynomials $R_{1}(z),\ldots ,R_{2p}(z)$ for any regular sequence $c_{-\varkappa }^{\varkappa }$. Similarly, we can define the left essential polynomials $L_{1}(z),\ldots ,L_{2p}(z)$ of the sequence $c_{-\varkappa }^{\varkappa }$.
  By theorem 3.1 from [14], the sequence $c_{-\varkappa }^{\varkappa }$ defined in equation (2.5) is regular and there exist respective essential polynomials $R_{1}(z),\ldots ,R_{2p}(z)$; $L_{1}(z),\ldots ,L_{2p}(z)$ such that
  (1) (i) the constant terms of the polynomials $R_{1}(z),\ldots ,R_{p}(z)$ are equal to zero,
  (2) (ii) the leading terms of the polynomials $L_{p+1}(z),\ldots ,L_{2p}(z)$ are equal to zero.
  **Definition 2.3.**
  The essential polynomials $R_{1}(z),\ldots ,R_{2p}(z);L_{1}(z),\ldots ,L_{2p}(z)$ satisfying the conditions (i)–(ii) are called the factorization essential polynomials of the sequence.
**Step 5. Construction of the factorization of the initial matrix polynomial, a(z).**
  Now we are in a position to deliver a final result on the explicit Wiener–Hopf factorization of a matrix polynomial a(z) (see theorem 3.2 in [14]). It is crucial to note here that, in contrast to any other methods, if it exists for a particular matrix function, our approach requires simultaneous consideration of both the right and the left factorizations.
  **Theorem 2.4.**
  *Let* $\mu_{1},\ldots ,\mu_{2p}$ *be the indices and* $R_{1}(z),\ldots ,R_{2p}(z)$ $\left(L_{1}(z),\ldots ,L_{2p}(z)\right)$ *are the right (left) factorization essential polynomials of the sequence* $c_{-\varkappa }^{\varkappa }$. *Let us introduce the* p×p *matrix functions*
  2.11 $$R_{1}(z)=\left(\begin{array}{c} R_{1}(z)\;\ldots \;R_{p}(z) \end{array}\right)\;\mathrm{and}\;L_{2}(z)=\left(\begin{array}{c} L_{p+1}(z)\\ \vdots \\ L_{2p}(z) \end{array}\right)$$
  *and*
  $$d_{L}(z)=\left(\begin{array}{ccc} z^{-\mu_{1}} & \ldots & 0\\ \vdots & \ddots & \vdots \\ 0 & \ldots & z^{-\mu_{p}} \end{array}\right)\;\mathrm{and}\;d_{R}(z)=\left(\begin{array}{ccc} z^{\mu_{p+1}} & \ldots & 0\\ \vdots & \ddots & \vdots \\ 0 & \ldots & z^{\mu_{2p}} \end{array}\right).$$
  *Then the left* ($\lambda_{1}\geq \cdots \geq \lambda_{p}$) *and right* ($\rho_{1}\leq \cdots \leq \rho_{p}$) *partial indices and the factors* ($l_{\pm }(z)$, $r_{\pm }(z)$) *of the respective factorizations of the matrix polynomial* a(z) *are defined by the formulae*
  2.12 $$\begin{array}{rl} & \lambda_{1}=-\mu_{1},\ldots ,\lambda_{p}=-\mu_{p}\;\rho_{1}=\mu_{p+1},\ldots ,\rho_{p}=\mu_{2p}, \end{array}$$
  2.13 $$\begin{array}{rl} & l_{-}(z)=z^{\varkappa +1}\Delta_{-}(z)d_{L}^{-1}(z)R_{1}^{-1}(z)\;l_{+}(t)=z^{-\varkappa -1}\Delta_{-}^{-1}(z)a(z)R_{1}(z) \end{array}$$
  2.14 $$\begin{array}{rl} \mathrm{and}\; & r_{-}(z)=\Delta_{-}(z)L_{2}^{-1}(z)\;r_{+}(z)=\Delta_{-}^{-1}(z)d_{R}^{-1}(z)L_{2}(z)a(z). \end{array}$$
  In the statement of this theorem, we have corrected the misprints appearing in the formulae for the factors $l_{+}(z)$, $r_{+}(z)$ in theorem 3.2 of [14].
  It follows from this theorem that the indices of the sequence $c_{-\varkappa }^{\varkappa }$, in addition to (2.9), satisfy conditions
  2.15 $$\sum_{j=1}^{p}\mu_{j}=-\varkappa \;\mathrm{and}\;\sum_{j=p+1}^{2p}\mu_{j}=\varkappa .$$

Those five steps above represent an *explicit* algorithm, since their implementation requires only using the linear algebra tools for objects of finite dimensions.

**Remark 2.5.**

The main difficulty in the practical implementation of the aforementioned formulae is that the sequence $c_{-\varkappa }^{\varkappa }$ can be found, in general cases, only numerically (and thus, the result is computed with some accuracy). Therefore, in such cases, the algorithm would produce the factorization of a matrix polynomial $\tilde{a}(z)$ close enough to the original matrix polynomial a(z) with all consequences of such replacement (possible instabilities as discussed above).

**Remark 2.6.**

If, by any means, one believes that the partial indices of a matrix polynomial in question are stable, some computations from Step 4 can be avoided (see example in §6).

## 3. An *exact* realization of the explicit algorithm

The analysis of the algorithm, carried out in the previous section, shows the reason for the instability of the factorization problem for matrix polynomials. The explanation is in the instability of the problems of finding the rank and null-space of a matrix. Since finding the rank is the key problem for our algorithm (and also for the Gohberg–Lerer–Rodman algorithm [15]), in the general case, an explicit algorithm cannot be implemented numerically.

However, if Steps (1)–(5) can be carried out in exact arithmetic, i.e. the error-free calculations are used, *problems with the factorization instability, with the accuracy of calculation and rounding errors do not arise.* It should be noted that symbolic calculations have the significant drawback that they often require significant computational resources. In our case, an increase in the size of the matrix function and the degree of the matrix polynomial can lead to a significant slowdown in the algorithm. Fortunately, in most of the applications, matrix functions of the second or third order mainly appear, and the only reason for a possible slowdown is a large degree of the matrix polynomial. In table 1, we illustrate this feature in detail.

**Table 1.** Execution time, $t_{k}$, (in seconds) for factorization of the matrix polynomial $C^{k}(z)$, defined in (5.4). The letter ‘F’ indicates a failure occurred during the execution, while the symbol ‘–’ highlights that the computations, performed in the symbolic mode, were unable to complete the task within 1 h.

						$t_{k}$ (symnum)		
k	$n_{k}$	$p_{k}$	$\varkappa_{k}$	$\varkappa_{2}\;(T_{0})$	$t_{k}$ (sym)	digits=5	digits=10	digits=15
1	4	6	3	1.10×10	0.28	0.4	0.31	3.44
2	8	12	6	7.95×10	0.81	1.0	0.61	0.62
3	12	18	9	$6.04\times 10^{2}$	2.62	1.4	1.77	1.80
4	16	24	12	$5.78\times 10^{3}$	7.19	F	2.94	2.95
5	20	30	15	$5.90\times 10^{4}$	23.78	F	7.95	8.83
6	24	36	18	$5.95\times 10^{5}$	55.11	F	10.66	13.26
7	28	42	21	$5.66\times 10^{6}$	147.23	F	35.80	38.11
8	32	48	24	$5.13\times 10^{7}$	288.67	F	44.89	42.19
9	36	54	27	$4.65\times 10^{8}$	644.03	F	F	135.55
10	40	60	30	$4.44\times 10^{9}$	1103.06	F	F	150.59
11	44	66	33	$4.53\times 10^{10}$	2204.62	F	F	453.78
12	48	72	36	$4.66\times 10^{11}$	—	F	F	444.05
13	52	78	39	$4.57\times 10^{12}$	—	F	F	F

The main result is formulated in theorem 3.2. It gives a necessary and sufficient condition for the *exact* factorization of a matrix polynomial a(z)∈Q(i)[z], relying on the same property of its determinant, deta(z). Below, we discuss a practical implementation of those five steps of the *explicit* algorithm in this particular arithmetic.

**Input data.** A matrix polynomial $a(z)=\sum_{j=0}^{n}a_{j}z^{j}$, $a_{j}\in C^{p\times p}$, of degree n that is invertible on the unit circle T. The entries of the matrix coefficients $a_{j}$ belong to the field Q(i).

**Step 1.** In order to construct the Wiener–Hopf factorization of the scalar polynomial deta(z) in *exact* arithmetic, it is sufficient to require that all roots of deta(z) belong to the field Q(i). *However, this condition is not necessary.* There exists a weaker necessary and sufficient condition allowing *exact* Wiener–Hopf factorization of deta(z) over the field Q(i). The idea here is to factorize the polynomial as a product of irreducible factors, testing (for example, with help of the Schur algorithm) whether the roots of each factor lie in the interior or exterior of the unit disk.

First, we give the criterion in terms of coefficients of the irreducible factors of the polynomial. As has been mentioned above, we assume that no zero lies on the unit circle. Note that, in the opposite case, the Schur test fails during the execution stage. This fact explains why we have not concentrated on how to verify the condition deta(z)≠0 on the first step of the algorithm.

Let $Q(z)=q_{0}+q_{1}z+\cdots +q_{N}z^{N}$ be a polynomial with complex coefficients, $q_{N}\neq 0$. We denote $Q^{*}(z)={\bar{q}}_{N}+{\bar{q}}_{N-1}z+\cdots +{\bar{q}}_{0}z^{N}$, where ${\bar{q}}_{j}$ is the complex conjugate of $q_{j}$.

For the given Q(z), we define the triangular Toeplitz matrices

$$U_{Q}:=\left(\begin{array}{ccc} q_{N} & \cdots & q_{1}\\ \vdots & \ddots & \vdots \\ 0 & \cdots & q_{N} \end{array}\right)\;\mathrm{and}\;L_{Q}:=\left(\begin{array}{ccc} q_{0} & \cdots & 0\\ \vdots & \ddots & \vdots \\ q_{N-1} & \cdots & q_{0} \end{array}\right)$$

and form the Hermitian N×N matrix $C_{Q}:=U_{Q}^{*}U_{Q}-L_{Q}L_{Q}^{*}$. Here, $U_{Q}^{*}$ ($L_{Q}^{*}$) is Hermitian conjugate of $U_{Q}$ ($L_{Q}$).

Let p(z) be a polynomial over the field Q(i). It can be represented as a product of irreducible monic polynomials [28]:

3.1 $$p(z)=p_{0}Q_{1}(z)\cdots Q_{m}(z),\;0\neq p_{0}\in Q(i).$$

**Theorem 3.1.**

*The polynomial* p(z) *admits the exact Wiener–Hopf factorization if and only if, for every polynomial* $Q_{j}(z)$, j=1,…,m, *one of the matrices* $C_{Q_{j}}$ *or* $C_{Q_{j}^{*}}$ *is positive definite*.

*Suppose these conditions are fulfilled. Denote by* $p_{1}(z)$ *the product of those irreducible factors* $Q_{j}(z)$ *in decomposition* (3.1) *for which the matrices* $C_{Q_{j}}$ *are positive definite. Then, the polynomial* $p_{2}(z)$ *is the product of the remaining irreducible factors and* $p_{0}$. *Let us define*: $\varkappa =\deg p_{1}(z)$, $p_{-}(z)=z^{-\varkappa }p_{1}(z)$ *and* $p_{+}(z)=p_{2}(z)$. *Then the exact Wiener–Hopf factorization of the polynomial* p(z) *is*

3.2 $$p(z)=p_{-}(z)z^{\varkappa }p_{+}(z).$$

**Proof.**

It is clear that the polynomial p(z) admits the exact Wiener–Hopf factorization if any of the irreducible factors has all its roots lying inside or outside the unit circle. By the criterion Schur–Cohn [29,30], the polynomial $Q_{j}(z)$ has all its roots lying inside the unit circle if and only if the matrix $C_{Q_{j}}$ is positive definite. Similarly, $Q_{j}(z)$ has all roots lying outside the unit circle if $Q_{j}^{*}(z)$ has all its roots lying inside the unit circle, that is $C_{Q_{j}^{*}}$ is positive definite. The representation (3.2) is the Wiener–Hopf factorization that is normalized by the condition $p_{-}(\infty )=1$.

For practical computation, it is more convenient to use the Schur test [31] instead of checking the positive definiteness of matrices. This test states (see, for example, [29]) that

*a polynomial $Q(z)=q_{0}+q_{1}z+\cdots +q_{N}z^{N}$ has all its roots lying inside the unit circle if and only if*

(i) $|q_{0}|<|q_{N}|$, and
(ii) the polynomial ${\tilde{Q}}_{1}(z)$ determined from the equality ${\tilde{Q}}_{1}(z)=z^{-1}({\bar{q}}_{N}Q(z)-q_{0}Q^{*}(z))$ possesses the same property.

Since $\deg{\tilde{Q}}_{1}(z)<\deg Q(z)$, then in a finite number of steps, we arrive at a polynomial of degree 0 or find that the polynomial Q(z) does not have the required property.

In order to verify that all roots of Q(z) lie outside the unit circle, it is necessary to apply the Schur test to the polynomial $Q^{*}(z)$. Note that the above algorithm automatically verifies the invertibility of the matrix polynomial a(z) on the unit circle T.

**Step 2.** Since the polynomial deta(z) admits the *exact* factorization $\det a(z)=\Delta_{-}(z)z^{\varkappa }\Delta_{+}(z),$ the coefficients of the polynomial $\Delta_{-}(z)$ in $z^{-1}$ belong to the field Q(i). The function $\Delta_{-}^{-1}(z)$ is analytic in the domain |z|>r, for some r<1. Its Laurent coefficients $\delta_{j}\in Q(i)$ are obtained by equation (2.4) in *exact* computations. Since only a finite number (2ϰ+1) of the coefficients are needed and all operations are arithmetic, Step 2 is therefore performed in the *exact* manner.

**Step 3.** The matrices $c_{-\varkappa },\ldots ,c_{\varkappa }$ defined in equation (2.5) have the entries belonging to Q(i), i.e. the sequence $c_{-\varkappa }^{\varkappa }$ is *exactly* computed. In Step 3 of the *explicit* algorithm, we find ranks and null spaces of block Toeplitz matrices $T_{k}(c_{-\varkappa }^{\varkappa })$ for −ϰ≤k≤ϰ. This step can be realized by Gaussian elimination. Since entries of the matrices belong to Q(i), the Gaussian elimination can be implemented in *exact* arithmetic [28].

**Step 4.** Obtaining the indices and the factorization essential polynomials of the sequence $c_{-\varkappa }^{\varkappa }$, defined in (2.8) and (2.11), is a foundation stone of the method. Since the ranks of the matrices $T_{k}(c_{-\varkappa }^{\varkappa })$ have been calculated *exactly*, the differences, $\Delta_{k}$, and hence the indices, $\mu_{j}$, are also found *exactly*.

To determine the essential polynomials, we need bases of the complements $H_{\mu_{j}+1}^{R}$, j=1,…,2p, (see equation (2.10) and definition 2.2) and the factorization essential polynomials (see definition 2.3). It is intuitively clear that these polynomials can be found *exactly* using the methods of linear algebra. However, a rigorous proof of this fact is rather complicated and requires a careful study of all steps of the algorithm for determination of the factorization essential polynomials. It will be presented in the separate work.

**Step 5.** The main result of this section is the following.

**Theorem 3.2.**

*Let* a(z) *be a matrix polynomial over the field of Gaussian rationales* Q(i). *Then* a(z) *admits* *exact* *Wiener–Hopf factorization if the polynomial* deta(z) *is exactly factorable over* Q(i).

**Proof.**

If a(z) admits the exact left Wiener–Hopf factorization $a(z)=l_{+}(z)d_{l}(z)l_{-}(z)$, then $\det a(z)=\det l_{+}(z)z^{\lambda_{1}+\cdots +\lambda_{p}}\det l_{-}(z)$ is the *exact* factorization of deta(z).

Conversely, if the polynomial deta(z) admits the *exact* factorization then all preliminary Steps 1–4 can be carried out exactly. It remains to use the formulae (2.12)–(2.14).

## 4. Implementation of the exact algorithm in Maple’s code: ExactMPF package

The *exact* factorization algorithm has been realized in the computer algebra system Maple using its linear algebra utilities as the package ExactMPF. The package can work in the version Maple 11 or higher. Clearly, one can do this in any other suitable software (for example, Mathematica^2^ ). The main procedure of the package is SolverExactMPF. During the execution, it calls a number of subroutines. We list the basic ExactMPF’s procedures:

— **SchurTest** verifies whether all polynomial roots lie in the unit circle (see §3, Step 1).
— **SolverExactPF** verifies whether a scalar polynomial admits the exact Wiener–Hopf factorization and builds it (see §3, Step 1).
— **ExactMLC** computes the matrix Laurent coefficients $c_{-\varkappa },\ldots ,c_{0},\ldots ,c_{\varkappa }$ for the rational matrix function $\Delta_{-}^{-1}(z)a(z)$ (see equation (2.5) and §3, Step 2).
— **seqTk** builds the sequence of the matrices $T_{k}(c_{-\varkappa }^{\varkappa })$, −ϰ≤k≤ϰ (§3, Step 3).
— **ExactFEP** finds indices and factorization essential polynomials of the sequence $c_{-\varkappa }^{\varkappa }$.

The main procedure SolverExactMPF finds the exact factorizations of a p×p Laurent matrix polynomial $a(z)=\sum_{k=-m}^{n}a_{k}z^{k}$. It also checks and validates the input entered into the procedure. The following validations are performed:

— are elements of the matrix a(z) polynomials in z, $z^{-1}$?
— are these polynomials over the field Q(i)?
— does deta(z) admit the exact Wiener–Hopf factorization?

If the validation fails, the program is interrupted.

To access ExactMPF use the commands.

> read("ExactMPF.txt");

> with(ExactMPF);

> with(LinearAlgebra);

To obtain the factorizations of a(z), we run the procedure SolverExactMPF with argument a(z).

lplus, dl, lminus, rminus, dr, rplus := SolverExactMPF(a):

The procedure SolverExactMPF returns the factors lplus, dl, lminus of the left factorization and the factors rminus, dr, rplus of the right factorization.

This procedure also automatically performs a few tests for the constructed factorization

(i) it finds the sum of the partial indices,
(ii) checks whether the factors $l_{+}(z),r_{+}(z)$ ($l_{-}(z),r_{-}(z)$) are matrix polynomials in z (in $z^{-1}$) and
(iii) whether ${\left(\Delta_{\pm }(z)\right)}^{-1}\det l_{\pm }(z)$, ${\left(\Delta_{\pm }(z)\right)}^{-1}\det r_{\pm }(z)$ are constants.

A very peculiar option symnum is also built into the procedure SolverExactMPF. It can be evaluated by the command:

lplus, dl, lminus, rminus, dr, rplus := SolverExactMPF(a,"symnum"):

In this mode, SolverExactMPF converts the matrices $T_{k}(c_{-\varkappa }^{\varkappa })$ to matrices with floating point entries to the precision given by the global variable digits. Then, SolverExactMPF uses the procedure NumRank included in the package ExactMPF.

**NumRank** finds a numerical rank of a N×M matrix A with float point entries. A good practice is to use a numerical rank, which is defined with the help of the SVD method [24,27]. Recall some necessary definitions.

Let A be a N×M matrix and $\sigma_{1}\geq \cdots \geq \sigma_{s}\geq 0$, s=min{N,M}, its singular numbers. In the case of exact computation, if rank A=r, then $\sigma_{r}\neq 0$ and $\sigma_{r+1}=\cdots =\sigma_{s}=0$. The number $k_{2}(A)=\sigma_{1}/\sigma_{r}$ is called *the spectral condition number* of the rectangular matrix A [27,32].

It is known that perturbations of the singular values are of the same order of magnitude as the perturbations of the matrix entries. Thus, computation of the singular values is a stable procedure and this property has led to the definition of a numerical τ-rank (see [24,27]).

A numerical τ-rank of the matrix function A(z) is the number of singular values greater than some tolerance τ, i.e. the singular values that are less than or equal to τ considered as perturbations of the zero singular values.

Thus, the tolerance τ specifies a way of separating the non-zero singular numbers from those assumed to be zero before the perturbation. Hence, the notion of τ-rank is useful when there exists a well-defined gap between non-zero and zero singular values of the matrix. Even for a matrix of the full rank, this requirement may not be fulfilled (if the matrix is ill-conditioned related to the spectral condition number). Choice of the parameter τ may be very sensitive and thus it is the most delicate and difficult task to decide upon in calculation of the numerical τ-rank. In the procedure **NumRank**, a default value of the tolerance is $\tau (A)=\max\{N,M\}\cdot \sigma_{1}(A)\cdot 10^{-\text{digits}}$.

Further, τ-ranks of the block Toeplitz matrices $T_{k}$ are used to define τ-indices of the sequence $c_{-\varkappa }^{\varkappa }$ instead of the *exact* indices of this sequence. All remaining calculations are performed in the *exact* arithmetic. If the output is validated successfully, the replacement of the indices by τ-indices may be considered as justified. Using this mode, one can significantly speed up the calculations. Moreover, this mode allows us to perform numerical experiments to test a coincidence of the partial indices and the partial τ-indices that were found by τ-indices of the sequence $c_{-\varkappa }^{\varkappa }$. In the next section, we perform such experiments.

After this short description of the ExactMPF package, we come back to matrix polynomials $P_{1}(z)$ and $P_{2}(z)$ discussed in the Introduction and try to factorize them *exactly* using the package.

**Example 4.1.**

Consider first the matrix function $a(z)=P_{1}(z)$ defined in (1.2) and execute

 > SolverExactMPF(a):

‘Failure: The exact polynomial factorization doesn’t exist’

‘The factorization process is interrupted’

**Example 4.2.**

Consider the second matrix polynomial $a(z)=P_{2}(z)$ from (1.2) that allows for the *exact* factorization of its determinant over the field Q(i). The procedure SolverExactMPF gives in this case the following expressions for the factors of a(z):

 > lplus; dl; lminus;

$$\left[\begin{array}{cc} -4z-\frac{1099}{116} & 2z-\frac{43}{29}\\ z^{2}+\frac{93}{58}z+\frac{48}{29} & \frac{2}{29}z+\frac{32}{29} \end{array}\right],\;\left[\begin{array}{cc} z & 0\\ 0 & z \end{array}\right]\;\mathrm{and}\;\left[\begin{array}{cc} -\frac{2}{29z} & 1-\frac{35}{58z}\\ 1+\frac{3}{29z} & \frac{1051}{232z} \end{array}\right],$$

 > rminus; dr; rplus;

$$\left[\begin{array}{cc} 0 & -4+\frac{2}{z}-\frac{1}{z^{2}}\\ \frac{35}{22} & \frac{3}{2}+\frac{3}{4z}+\frac{4}{z^{2}} \end{array}\right],\;\left[\begin{array}{cc} 1 & 0\\ 0 & z^{2} \end{array}\right]\;\mathrm{and}\;\left[\begin{array}{cc} \frac{44}{35}+\frac{121}{140}z+\frac{33}{70}z^{2} & \frac{11}{70}z-\frac{11}{35}z^{2}+\frac{22}{35}z^{3}\\ -\frac{1}{2} & 1 \end{array}\right].$$

The execution time is 0.265 s.

Note that the left and right partial indices do not coincide. Moreover, the set of partial indices in the left factorization is stable, while that for the right factorization is unstable. In the next section, we carry out more extensive numerical experiments with ExactMPF.

## 5. Examples of matrix factorization using the ExactMPF package

All computations have been performed on a home desktop computer HP with Intel(R) Core(TM)i3-415T CPU, 3.00 GHz, 4G RAM, operating the system Windows 10.

The package ExactMPF can be used for study of the *explicit* factorization of matrix polynomials. In the electronic supplementary material, we include numerous tests performed with use of the ExactMPF package, containing solutions of all examples from the main body of the paper.

### (a) Factorization of triangular matrices

First, we consider factorization of triangular matrix functions. In [33], the right factorization of triangular 2×2 matrix functions A(t) is studied in detail and the stability analysis of this problem is carried out. It has been proven that, for a non-trivial case, the factorization of A(t) is reduced to the factorization of the following Laurent matrix polynomial

5.1 $$P(t)=\left(\begin{array}{cc} t^{\nu_{1}} & 0\\ \sum_{j=\nu_{1}+1}^{\nu_{2}-1}a_{j}t^{j} & t^{\nu_{2}} \end{array}\right),$$

where $\nu_{2}\geq \nu_{1}+3$. If $a_{j}\in Q(i)$, factorization of such a matrix can be naturally constructed with the help of our package.

**Example 5.1.**

Consider

$$P(z)=\left(\begin{array}{cc} \frac{1}{z^{3}} & 0\\ -\frac{1}{z^{2}}-\frac{4}{5z}+1-z^{2}+z^{3} & z^{4} \end{array}\right).$$

Then, after execution of SolverExactMPF, we get

 > rminus, dr, rplus;

$$\begin{array}{rl} & \left[\begin{array}{cc} 1+\frac{4}{z}+\frac{21}{5z^{2}}+\frac{16}{5z^{3}} & \frac{1}{z^{2}}+\frac{1}{z^{3}}+\frac{1}{z^{4}}\\ -\frac{104}{25}-\frac{169}{25z}-\frac{16}{5z^{2}} & \frac{1}{5}-\frac{4}{5z}-\frac{9}{5z^{2}}-\frac{1}{z^{3}} \end{array}\right]\;\left[\begin{array}{cc} 1 & 0\\ 0 & z \end{array}\right]\\ \mathrm{and}\; & \left[\begin{array}{cc} -5z & -5z^{2}-5z-5\\ 5z^{2}+15z+1 & 5z^{3}+20z^{2}+21z+16 \end{array}\right], \end{array}$$

 > lplus, dl, lminus;

$$\left[\begin{array}{cc} 0 & 1\\ 1 & -z-\frac{4}{5}z^{2}+z^{3}-z^{5}+z^{6} \end{array}\right],\;\left[\begin{array}{cc} z^{4} & 0\\ 0 & z^{-3} \end{array}\right]\;\mathrm{and}\;\left[\begin{array}{cc} 0 & 1\\ 1 & 0 \end{array}\right].$$

The execution time is 0.547 s. We note that in this case, the left factorization can be easily constructed without the package, but with another order for the left partial indices

$$\left[\begin{array}{cc} 1 & 0\\ -z-\frac{4}{5}z^{2}+z^{3}-z^{5}+z^{6} & 1 \end{array}\right]\;\left[\begin{array}{cc} z^{-3} & 0\\ 0 & z^{4} \end{array}\right]\;\mathrm{and}\;\left[\begin{array}{cc} 1 & 0\\ 0 & 1 \end{array}\right].$$

As is clear from the presented example, the indices of the diagonal elements in the triangular matrix do not always coincide with its partial indices [33].

The factorization of triangular matrix functions of an arbitrary order requires considerations of a number of special cases, depending on the relation between the indices of the diagonal elements [34]. The procedure SolverExactMPF can be useful for experimenting with such matrices.

**Example 5.2.**

Let

$$A(z)=\left(\begin{array}{ccc} z-\frac{1}{2} & 0 & 0\\ 0 & \frac{z^{2}\;+\;13z\;+\;15}{z^{2}} & 0\\ z-1 & z^{-1} & 1 \end{array}\right).$$

Here, the indices of diagonal elements are $\varkappa_{1}=1$, $\varkappa_{2}=-2$, $\varkappa_{3}=0$. SolverExactMPF returns the following data:

 > lplus, dl, lminus;

$$\left[\begin{array}{ccc} 2 & 1 & 0\\ 0 & 0 & z^{2}+13z+15\\ 2 & 2 & z \end{array}\right],\;\left[\begin{array}{ccc} z & 0 & 0\\ 0 & 1 & 0\\ 0 & 0 & z^{-2} \end{array}\right]\;\mathrm{and}\;\left[\begin{array}{ccc} \frac{1}{2} & 0 & -\frac{1}{2z}\\ -\frac{1}{2} & 0 & 1\\ 0 & 1 & 0 \end{array}\right]$$

Since the expression for the right factors $r_{\pm }(z)$ are cumbersome (see the electronic supplementary material for full details), we present only the middle factor

 > dr

$$\left[\begin{array}{ccc} z^{-1} & 0 & 0\\ 0 & z^{-1} & 0\\ 0 & 0 & z \end{array}\right].$$

The executing time is 0.563 s.

Again, both the left and right partial indices of this matrix function do not coincide with the indices of the diagonal elements.

### (b) Factorization of a matrix polynomial with complex coefficients

In the following example, we construct the *exact* factorization of a matrix polynomial with complex coefficients.

**Example 5.3.**

$$B(z)=\left(\begin{array}{cc} 2iz^{2}-z+1/2-z^{-1} & -1\\ z^{2}-iz+2+4iz^{-1} & z^{3}-iz^{2}+2z+4i \end{array}\right).$$

Then, we get

 > lplus, dl, lminus, dr;

$$\left[\begin{array}{cc} 2i & -1\\ 0 & z^{3}-iz^{2}+2z+4i \end{array}\right]\;\left[\begin{array}{cc} z^{2} & 0\\ 0 & 1 \end{array}\right],\;\left[\begin{array}{cc} 1+(i/2)z^{-1}-(i/4)z^{-2} & 0\\ z^{-1} & 1 \end{array}\right]\;\mathrm{and}\;\left[\begin{array}{cc} z & 0\\ 0 & z \end{array}\right].$$

The execution time is 0.438 s.

For the same reason as above, we present the factors only for the left factorization.

### (c) Experiments with the τ-factorization of matrix polynomials

As have been already underlined, the main unsolved problem in the approximate factorization of matrix polynomials is a verification whether its partial τ-indices coincide with the partial indices of the original matrix (both left and right ones). Since it has been impossible so far to prove any definite result theoretically, we carry out here numerical experiments using our designed solver SolverExactMPF in its symbolic-numeric mode symnum for the cases when exact factorization can be performed.

In the next example, we introduce a sequence of *exact* factorizable matrix polynomials with increasing degrees. We use the full capability of SolverExactMPF in both its modes (exact and symbolic-numeric) to calculate their partial indices and the partial τ-indices (see §4). Then we carry out all subsequent calculations in the *exact* arithmetic. If the obtained factors $l_{\pm }(z)$, $r_{\pm }(z)$ and the partial indices pass all the necessary verifications, then the factorizations are constructed correctly and the replacements of the ranks by their numerical τ-ranks is justified. Moreover, using option symnum mode gives an additional bonus: it significantly speeds up the computations.

The purpose of our experiments is to test applicability of the notion of the τ-rank for the factorization of matrix polynomials. *A priori*, it is clear that the method is ineffective if the task of computation of the τ-ranks for the sequence of block Toeplitz matrices $T_{-\varkappa },\ldots ,T_{0},\ldots ,T_{\varkappa }$ is extremely ill-conditioned. Let us define an analogue of the condition number for this task.

As usual, if A is a rectangular matrix, then $\varkappa_{2}(A)=\sigma_{1}/\sigma_{r}$, r=rankA, is the condition number of A with respect to the spectral matrix norm. We say that $K_{2}=\max_{-\varkappa \leq j\leq \varkappa }\varkappa_{2}(T_{j})$ is the condition number of the matrix sequence ${\left\{T_{j}\right\}}_{j=-\varkappa }^{\varkappa }$.

Since the τ-rank can be found only numerically, we are able only to estimate the value of $K_{2}$. It is easy to verify that $\text{rank}\;T_{0}=\varkappa (p-1)+p$. Hence, we can explicitly calculate $\varkappa_{2}(T_{0})$ and can find a lower bound for $K_{2}\geq \varkappa_{2}(T_{0})$. As has been mentioned above, a choice of parameter τ, to be used in τ-factorization, is not trivial. We use in our analysis the value $\tau (T_{j})=\text{maxsize}(T_{j})\cdot \sigma_{1}(T_{j})\cdot 10^{-\text{digits}}$ as it is the same as the default tolerance in Matlab’s rank command. We can prove that

5.2 $$\begin{array}{rl} & \text{maxsize}(T_{j})\leq (2\varkappa +1)p,\;\sigma_{1}(T_{j})=||T_{j}|{|}_{F}\leq ||T_{\varkappa }|{|}_{F}=\sqrt{|c_{-\varkappa }{|}^{2}+\cdots +|c_{\varkappa }{|}^{2}},\\ & \;\;-\varkappa \leq j\leq \varkappa , \end{array}\}$$

where $||\cdot |{|}_{F}$ is the Frobenius norm of a matrix.

Taking into account (5.2), we see that

5.3 $$\tau \leq (2\varkappa +1)p\sqrt{|c_{-\varkappa }{|}^{2}+\cdots +|c_{\varkappa }{|}^{2}}\cdot 10^{-\text{digits}},$$

chosen as the tolerance τ is controlled by the value of parameter digits.

In the next example, we will see how the correctness of such an approach depends on the choice of the parameter digits.

**Example 5.4.**

The matrix polynomial

5.4 $$C(z)=\left(\begin{array}{cc} 5iz^{2}-3z+1 & -10iz^{2}+6z-2\\ z & z^{4}+z^{3}(-1-i)+2z^{2}-\frac{13z}{2} \end{array}\right)$$

admits the exact factorization. Let us consider matrix polynomials $C_{k}(z)=C^{k}(z)$ and denote $n_{k}=\deg\;C_{k}(z)$, $p_{k}=\deg\;\det\;C_{k}(z)$, $\varkappa_{k}=\text{ind}\;\det\;C_{k}(z)=k\varkappa$. We also record the execution time, $t_{k}$, required by the package to compute factorization of the matrix polynomial $C_{k}(z)$. Three different machine precisions: digits=5, 10 or 15 are used for the testing.

The results of the numerical experiment are shown in table 1. Expressions for the factors $l_{\pm }(z)$, $r_{\pm }(z)$ are very cumbersome and are omitted.

The test has demonstrated that the symbolic part of the ExactMPF package is able to deliver the results in reasonable time for 1≤k≤11 and the matrix polynomials $C_{k}(z)$ have the stable sets of their partial left and right indices. Those are

5.5 $$\begin{array}{rl} & \lambda_{1}=\lambda_{2}=\rho_{1}=\rho_{2}=\frac{k\varkappa }{2},\;k\;\text{is even},\\ \; & \lambda_{1}=\rho_{2}=\frac{k\varkappa +1}{2},\;\lambda_{2}=\rho_{1}=\frac{k\varkappa -1}{2},\;k\;\text{is odd}. \end{array}\}$$

From table 1, we can also see that the executions in the sym-mode, when the ranks are calculated in *exact* arithmetic, become very slow for large values of k and the use of symbolic-numeric calculation (symnum-mode) can greatly accelerate the computations.

In the cases indicated by the letter F in table 1, the procedure NumRank has found the ranks of $T_{k}$ incorrectly (the validation of the sum of the indices for the sequence $c_{-\varkappa }^{\varkappa }$ fails), and the ExactMPF package interrupts computations because of the failure. The reason for this is that the condition number $K_{2}$ was too large. In this case, increasing parameter digits (that decreases the threshold for numerical tolerance, τ) makes it possible to continue correct calculations. Note that the partial indices computed for k=12 with use of the τ-factorization follow the same rule as in (5.5). Unfortunately, this fact does not formally allow us to conclude (with 100% confidence) that the *exact* partial indices would be the same.

The next example shows that symnum mode is able to correctly calculate the partial indices even in unstable cases.

**Example 5.5.**

Let us consider a matrix polynomial

$$A=\left(\begin{array}{cc} 36z^{2}+17z-14 & 0\\ -z^{2}+3z & z^{2}+13z+15 \end{array}\right).$$

The *exact* computations using SolverExactMPF give $\lambda_{1}=\lambda_{2}=1$ and $\rho_{1}=0$, $\rho_{2}=2$. Moreover, in this case, $\varkappa_{2}(T_{0})=5.294083$.

Next, let us construct another matrix polynomial $B=A^{8}$. For this one, SolverExactMPF returns the following values of the partial indices: $\lambda_{1}=14$, $\lambda_{2}=2$ and $\rho_{1}=0$, $\rho_{2}=16$. In the symnum mode with the parameter digits=10, the package was unable to find the correct values since the tests on the sums of indicies are not successful and the factorization process is interrupted. In this case, $\varkappa_{2}(T_{0})=6.465447\times 10^{10}$ and, as a result, the condition number $K_{2}\geq 6.465447\times 10^{10}$ is too large. If, however, we set digits = 15, the package returns, in the symnum mode, the correct values of the partial indices.

Thus, our numerical experiments with several matrix polynomials (see electronic supplementary material) have shown that, for the case of not too large condition numbers, calculations with use of both the symbolic and numeric options of the ExactMPF package give identical partial indices and partial τ-indices, even in the unstable cases. This fact gives hope for a stable construction of an approximate factorization but requires a solid theoretical proof.

## 6. On numerical realization of the explicit algorithm

In previous sections, we considered the problem of the Wiener–Hopf factorization for matrix polynomial over the field Q(i). In this case,

(i) the input data are given with infinite precision, and
(ii) there is a possibility to use the *exact* (error-free) calculations.

As a result, we have constructed the ExactMPF package for the *exact* solution of the factorization problem, that returns the solution under condition that the determinate of the matrix polynomial is *exactly* factorizable or, in the opposite case, interrupts the computations. In this case, the stability and accuracy problems do not arise. However, a natural question arises: can we deliver an approximate (numerical) factorization of such a matrix? If the conditions for the existence of the exact factorization are not fulfilled, then it is not yet possible to build any algorithm for the factorization problem that satisfies the entire canon of computational mathematics (with carrying stability analysis, obtaining forward/backward errors and accuracy for approximate factorization).

Here, we highlight possible obstacles when implementing the *explicit* algorithm (Steps 1–5) numerically *without relating the steps to the ExactMPF* package, in the case, when, at least, one of the conditions (i), (ii) is not fulfilled.

**Input data.** We consider a matrix polynomial a(z) of the degree n and size p×p that is invertible on the unit circle T. The entries of the matrix coefficients, $a_{j}$, belong to the field C. They can be represented by floating point numbers with the accuracy $10^{-q}$ (finite precision) or the entries belong to the field Q(i) (infinite precision).

**Step 1.** Since one of the conditions (i), (ii) is not fulfilled, we can solve the factorization problem for the function Δ(z)=deta(z) only approximately. If the input data are given with infinite precision we convert them in the floating point format with a desired accuracy $10^{-q}$.
  A standard way for the factorization of a scalar polynomial, recommended by all explicit methods, suggests using approximate values of the polynomial roots. However, those roots are, in general, not well-conditioned functions of polynomial coefficients (see, e.g. [35]), and vice versa, the coefficients of a polynomial are also not well-conditioned functions of their roots [36]. The latter means that searching numerically for zeros of the polynomial to construct its factorization (2.1) may lead to the algorithm instability.
  In order to solve the factorization problem for scalar polynomials over the field C, a dedicated package PolynomialFactorization (in the Maple Software) has been developed by Adukov [37]. It factorizes an input polynomial p(z) based on the indices and essential polynomials technique. Input data for its main procedure SolverPF is a scalar polynomial p(z) and the accuracy $10^{-q}$ of the polynomial coefficients. SolverPF returns factors $p_{-}(z)$, $p_{+}(z)$ of the Wiener–Hopf factorization of p(z) and their *guaranteed* accuracy ε. The accuracy, ε, calculated by the procedure SolverPF depends on a type of polynomial and the accuracy of the input data.
  Note, that the PolynomialFactorization package is not included in the ExactMPF package, and is different from the standard ‘Factoring a Polynomial’ Maple command Factor.^3^ Its main feature is: it does not rely on computation of the polynomial’s roots.
**Step 2.** Now we can calculate the Laurent coefficients $\delta_{j}$ of $\Delta_{-}^{-1}(z)$ using given matrix coefficients $a_{j}$ of a(z) and taking into account their accuracy.
  Hence, we can find the sequence $c_{-\varkappa },\ldots ,c_{\varkappa }$ with some accuracy as described in §2. This step does not contain any technical difficulties. Note that, if the input data are given with infinite precision, i.e. belong to the field Q(i), then we can calculate $c_{j}$ with any reasonable accuracy. Otherwise, the accuracy is restricted by finite accuracy of input data.
**Step 3.** This is a crucial point in the factorization problem. In the *exact* arithmetic, the respective calculations do not meet any problem (except of computational time). However, if the entries of the matrices are known only approximately, evaluations of the ranks and null spaces of $T_{k}$ might be unstable. This is a reason for the instability of the factorization problem itself.
  In §4, we have discussed the procedure **NumRank** from ExactMPF in detail. It uses the SVD method for finding a numerical rank of matrices with floating point entries. The procedure can also be used in this case.
  For definition of the essential polynomials, we need a method for evaluation of a basis of the null space for matrices with floating point entries. This can also be done with the SVD method [24,27]. Computation practice shows that this method is the most reliable way to numerically find the rank and the basis for the null space of a matrix. Here, however, the main problem is how to select the computational tolerance τ. Unfortunately, in general, there is no rigorous way to do this. As an initial choice, we can recommend using $\tau (A)=\max\{N,M\}\cdot \sigma_{1}(A)\cdot 10^{-\text{digits}}$, if one works in the Maple environment.
**Step 4.** Since entries of the matrices $T_{k}$ (−ϰ≤k≤ϰ) are, in general, floating point numbers, we need to compute the numerical τ-rank of the sequence $c_{-\varkappa }^{\varkappa }$ instead of *exact*. The indices of the sequence $c_{-\varkappa }^{\varkappa }$ found by τ-ranks are respectively called τ-*indices*.
  Next, to construct an approximate Wiener–Hopf factorization of the matrix polynomial, we need to find the factorization essential polynomial of the sequence $c_{-\varkappa }^{\varkappa }$. In §3, we describe an algorithm for construction of these polynomials and prove that, for input data with infinite precision, the algorithm can be realized in the exact arithmetic. An open problem, however, is how to adapt the algorithm for input data with a finite precision. In such cases, we may use a basis of null space for matrix $T_{k}$ (−ϰ≤k≤ϰ) via the SVD method with the tolerance τ (τ-bases). The factorization essential polynomials, constructed with the help of τ-bases, are called factorization essential τ-polynomials.
**Step 5.** For the fixed tolerance τ>0, the integers $\lambda_{1}^{\tau },\ldots ,\lambda_{p}^{\tau }$, $\rho_{1}^{\tau },\ldots ,\rho_{p}^{\tau }$, computed by formulae (2.12) with the help of τ-indices of the sequence $c_{-\varkappa }^{\varkappa }$ are called partial τ-indices of the matrix polynomial a(z). The factorization of a(z) constructed by formulae (2.12)–(2.14) with the help the partial τ-indices and the factorization essential τ-polynomials will be called τ-factorization of the matrix polynomial a(z).

The key question here remains open:

*Does the τ-factorization of a matrix polynomial a(z) with a chosen τ represent an approximate factorization of a(z) (preserving the same set of the partial indices) with a certain accuracy?*

To solve this problem, we must find answers to the following basic questions:

(i) does a tolerance τ exist such that the partial τ-indices of a(z) coincide with the matrix partial indices? How can this tolerance be found?
(i) can the algorithm for constructing factorization essential polynomials from §3 be adapted to find the factorization essential τ-polynomials?

In order to carry out numerical experiments, we should develop a new appropriate software. At the present time, we have in our hands the package PolynomialFactorization from [37] and the procedure NumRank from this ExactMPF package. Since the procedure for finding the factorization essential τ-polynomials is still unavailable, in the following example, we restrict ourselves to the evaluation of partial τ-indices only.

We consider the matrix polynomial $P_{1}(z)$ from the Introduction in (1.2). Note that the input data have infinite precision, while the determinant $\det P_{1}(z)$ can be factorized only approximately. Let us first construct a factorization of $p(z)=\det P_{1}(z)$ with the help of the package PolynomialFactorization to obtain

$$p_{1}(z)=1.00000000z^{2}-0.4572987z+0.1301582$$

and

$$p_{2}(z)=2.0000000z^{3}+1.9145973z^{2}+5.8652264z+7.6829597,$$

with a precision ϵ=0.0000034. This precision was returned by the package PolynomialFactorization proceeded under the Maple value of digits=15 and accuracy $10^{-q}=10^{-15}$.

Thus, an approximate factorization (2.1) has been constructed with the index $\varkappa =\det P_{1}=\deg p_{1}=2,$ and the factors $\Delta_{-}(z)=z^{-2}\;p_{1}(z)$ and $\Delta_{+}(z)=p_{2}(z).$

The Laurent coefficients of $\Delta_{-}^{-1}(z)$ are found directly from the recurrence formulae (2.4)

$$\begin{array}{rl} & \delta_{0}=1,\;\delta_{1}=0.4572986,\;\delta_{2}=0.0789639,\delta_{3}=-0.0234111,\\ & \;\delta_{4}=-0.0209836\;\mathrm{and}\;\delta_{5}=-0.0065486. \end{array}$$

Calculation of the matrix Laurent coefficients by formulae (2.5) gives the following result:

$$c_{-2}=\left(\begin{array}{cc} 0.001184 & -0.041851\\ -0.023411 & 0.264908 \end{array}\right)\;\mathrm{and}\;c_{-1}=\left(\begin{array}{cc} -0.011461 & -0.205726\\ 0.078963 & 1.863760 \end{array}\right)$$

and

$$\begin{array}{rl} & c_{0}=\left(\begin{array}{cc} -0.049372 & -0.401256\\ 0.457298 & 4.512850 \end{array}\right),\;c_{1}=\left(\begin{array}{cc} -0.085404 & 0.170808\\ 1.000000 & 1.536260 \end{array}\right)\\ \mathrm{and}\; & c_{2}=\left(\begin{array}{cc} 2.000000 & -4.000000\\ 0.000000 & 1.45730 \end{array}\right). \end{array}$$

In order to verify the stability criterion for matrix polynomials (theorem 3.1 in [23]), we need to compute the τ-ranks of matrices

$$T_{-1}=\left(\begin{array}{cc} c_{-1} & c_{-2}\\ c_{0} & c_{-1}\\ c_{1} & c_{0}\\ c_{2} & c_{1} \end{array}\right)\;\mathrm{and}\;T_{1}=\left(\begin{array}{cccc} c_{1} & c_{0} & c_{-1} & c_{-2}\\ c_{2} & c_{1} & c_{0} & c_{-1} \end{array}\right).$$

This can be done with use of the function SingularValues(A) of the MapleSoftware that returns the singular values of a matrix A. For the 8×4 matrix $T_{-1}$, it gives the following singular values:

$$\begin{array}{rl} & \sigma_{1}=7.449916,\;\sigma_{2}=4.432173,\;\sigma_{3}=1.349779,\\ & \sigma_{4}=0.737172\;\mathrm{and}\;\sigma_{5}=\sigma_{6}=\sigma_{7}=\sigma_{8}=0.0. \end{array}$$

and, therefore, $\tau (T_{-1})\approx 6.0\times 10^{-14}$ for digits=15.

Since the gap between $\sigma_{4}$ and $\sigma_{5}$ is much larger than the above tolerance, we can conclude that τ-$\text{rank}(T_{-1})=4$. Since $k_{2}(T_{-1})=\sigma_{1}/\sigma_{4}\approx 10.11$, it appears that $T_{-1}$ is a well-defined matrix of the full τ-rank.

Similarly, the 4×8 matrix $T_{1}$ has the following non-zero singular values:

$$\sigma_{1}=7.052308,\;\sigma_{2}=4.295688,\;\sigma_{3}=3.050511\;\mathrm{and}\;\sigma_{4}=0.130922,$$

while $\tau (T_{1})\approx 5.64\times 10^{-14}$ and $k_{2}(T_{1})\approx 53.87$. Hence, $T_{1}$ also is a well-defined matrix of the full τ-rank.

Let us represent $\varkappa =\text{ind}\det P_{1}$ in the form ϰ=sp+r, where s and 0≤r<p are integers. In this case ϰ=p=2, i.e. s=1, r=0. Then, by the above-mentioned theorem 3.1 from [23], the left (right) partial indices of $P_{1}(z)$ are stable if and only if $\text{rank}\;T_{-s}=(\varkappa +1)p-\varkappa (\text{rank}\;T_{s}=(\varkappa +1)p-\varkappa )$, that is: $\text{rank}\;T_{-1}=4$ ($\text{rank}\;T_{1}=4$). Thus, we can conclude that τ-indices are $\lambda_{1}^{\tau }=\lambda_{2}^{\tau }=1$ and $\rho_{1}^{\tau }=\rho_{2}^{\tau }=1$. Finally, since the matrices $T_{-1}$, $T_{1}$ are well-conditioned, *we can expect* that the partial indices of $P_{1}(z)$ are also stable and equal to those computed numerically. But this needs anyway a rigorous proof.

## 7. Conclusion

We have used the *explicit* algorithm for the *exact* Wiener–Hopf factorization of a matrix polynomial a(z) with rational coefficients and with exact factorizable deta(z) and described the main steps in its realization within the ExactMPF package. Its listing is included into the electronic supplementary material.

A message delivered by this paper is twofold: (a) the existence of an *explicit* algorithm does not necessarily guarantee that the factorization can be effectively performed; and, on the other hand, (b) in contrast to the general opinion, it is indeed possible to find sometimes *exact* values of the partial indices and then to perform *exact* factorization, even though the indices are not stable.

One of the main results of this paper is the necessary and sufficient condition for existence of the *exact* factorization for matrix polynomials whose coefficients are matrices with entries from the Gaussian rational field Q(i). It turns out that a matrix polynomial a(z) admits the *exact* Wiener–Hopf factorization if and only if the polynomial deta(z) is *exactly* factorable.

The second (and extremely useful) result of the paper is the development of the package ExactMPF in the Maple software that realizes factorization algorithm *exactly* for a matrix polynomial with the coefficients from the Gaussian rational field Q(i) and the exact factorizable determinant.

The use of the ExactMPF package is manyfold. The authors confidently declare that

— it can be used for symbolic or symbolic-numeral factorization of matrix polynomials.
— to analyse stability of the partial indices of matrix polynomials.
— to make tests and experiments for verification of proved statements and for a motivation to formulate hypothesis or conjectures for further theoretical development of the theory.

They also believe that it may be useful with additional efforts

— in some cases to build an approximate factorization of a given matrix polynomial with arbitrary complex coefficients and
— to solve real life problems as a computational tool. We shall demonstrate this in the accompanying paper dealing with solving of a discrete analogue of nonlinear Schrödinger equation by the inverse scattering transform method.

## Data Availability

The data are provided in electronic supplementary material [38].

## Footnotes

1 https://www.maplesoft.com/.

2 https://www.wolfram.com/mathematica/.

3 https://www.maplesoft.com/support/help/maple/view.aspx?path=Task/FactoringAPolynomial.
